# Supplementary material for: Development of a Honey Bee RNA Virus Vector Based on the Genome of a Deformed Wing Virus
Source: Viruses. 2020 Mar 28;12(4):374. doi: 10.3390/v12040374 (PMC7232236; doi:10.3390/v12040374)
Supplement: Supplementary file 1 [file viruses-12-00374-s001.zip › Text-S1_Design of GFP-tagged-DWV.pdf]

Text S1. Design of pDWV-L-GPF and DWV-S-GFP

The full-length infectious cDNA clone of DWV type A, pDWV-304 (GenBank accession number MG831200) [Rybov et al., 2019] was used to design eGFP-expressing viral constructs, pDWV-L-GFP and pDWV-S-GFP (Figure 1; Text S2). Cloning included the following steps. At the first stage, two DNA fragments corresponding to the nucleotide positions 1365 – 5089 of DWV-304 with the insertion of the sequence containing restriction sites *AscI* and *BamH*I at the LP-VP2 were generated by overlap extension PCR using the plasmid pDWV-304 as a template and the primers listed in Table S1. Both fragments had *Asc*I-*Bam*HI sequences inserted upstream the sequence coding for the proposed proteolytic cleavage peptide (VQAKPEMDNPNPG) between the LP and VP2 of DWV-304. Towards the 5' end from the *Asc*I site, the sequence coding for entire proposed proteolytic cleavage peptide at the DWV LP-VP2 interface (VQAKPEMDNPNPG) was inserted in the fragment destined for the construct pDWV-L-GFP, and the sequence coding for the proposed carboxy-terminal peptide of the DWV LP (VQAKPE) was inserted for fragment used in designing pDWV-S-GFP. As a result, the *Asc*I-*Bam*Hi sequence was linked with the sequences coding for the potential DWV LP-VP2 proteolytic cleavage peptides (Figure S1). For the pDWV-L-GFP design, the this fragment was generated by overlap extension PCR using mutagenic primers "14-AscBiamhi-VQAKPEM-For" and "15-MDNPNPG-BamhiAsci-Rev" and the flanking primers "1-Flank-For" and "2-Flank-Rev". For the pDWV-S-GFP design, the fragment was generated using mutagenic primers "14-AscIBamhi-VQAKPEM-For" and "16-VQAKPE-BamhiAsci-Rev", and the flanking primers "1-Flank-For" and "2-Flank-Rev". The resulting fragments were cloned into pCRII-TOPO vector (Invitrogen) and were used to insert the *Asc*I-*Bam*HI-flanked eGFP PCR fragment generated using the primers "17-Asci-GFP-for" and "18-BamH-GFP-Rev" and pAcP(+)IE1-eGFP [Shi et al., 2007] as a template to make clones with two types of the LP-eGFP-VP2 insert. Then *Sal*I-*Sac*II section of these DWV-LP-eGFP-VP2 clones were used to replace *Sal*I-*Sac*II section of the pDWV-304 (positions 1438-4273) to produce pDWV-L-GFP and pDWV-S-GFP (Fig. 1).

References:

Ryabov *et al.* (2019) Dynamic evolution in the key honey bee pathogen deformed wing virus: Novel insights into virulence and competition using reverse genetics. PLoS Biology 17, e3000502. DOI: https://doi.org/10.1371/journal.pbio.3000502

Shi, X. *et al.,* (2007). Construction and characterization of new piggyBac vectors for constitutive or inducible expression of heterologous gene pairs and the identification of a previously unrecognized activator sequence in piggyBac. *BMC Biotechnology* 7, 5. https://doi.org/10.1186/1472-6750-7-5
