# Supplementary material for: Development of a Honey Bee RNA Virus Vector Based on the Genome of a Deformed Wing Virus
Source: Viruses. 2020 Mar 28;12(4):374. doi: 10.3390/v12040374 (PMC7232236; doi:10.3390/v12040374)
Supplement: Supplementary file 1 [file viruses-12-00374-s001.zip › Text-S2__DWV-L-GFP-DWV-S-GFP-Sequences.pdf]

Text S2. Nucleotide sequences of the Deformed wing virus vector cDNA clones pDWV-L-GFP and pDWV-S-GFP. The clones are based on the clone DWV-304 (GenBank Accession number MG831200). GFP RNA inserts are shown in green, yellow highlight – duplicated flanking sequences derived from the DWV LP-VP2 border, blue highlight – sequences of *Asc*I and *Bam*HI restriction sites.

>DWV-L-GFP

TTTAAAATTCGCTATGGGAGGCGATTTATGCCTTCCATAGCGAATTACGGTGCAACTAACAATTTTAGATA
GTAGCCATGAACAAACATTATAGTGGCTCACTACGTATTGATCATTTTTATAATGACTTGCGTAGCATGAA
GCGCATGCTTGTAGTTGTAACTATGTTACTTTGCAAGTTGGAGTTTACTATTTTGGATTATGAATATGTGC
ACTTAGTGTCTGTATTTATAGTCGTTTGTGGTTCAAGGTTTTGTGTTAGTAGTACACTTATGTATGAATGT
ACCTTTAGTATGAATGTTATAGAATGACAATATCGAAAGAAAAATCTTTATAAAATACAAAAATATTGTTT
TTATTATTTCGATATGGTGTTTTATAGAGTAGATTGCCATGTGACCGCTCGTAGAAGTCCATTATGGTTTA
TCAATCGAAGTTGAATGTATTTATAAGAATATTATACTTAATTAATAATATTAGTAGTCCGTAACTATTAT
CATCCTTTTTCAGTTTGATGTGATAATAGACCACTGCAGTATCGAGTAGAGTTTCGAATGCGTAGTGCAAT
AGTACAATCACTGTCACCGACCATCTATTGTAATGATAGATTTGTCGGAAACCATTATTTATGAAGTGACT
AGCAATCATGGATTAAATTAGATGGTATTCTAGTTTAGAGGTGATTCGGCGCTGCGGTGCGACTGAAACTT
CTAAATTAGCATGTCAGATTGTATTATGAATGCGTTAGTAGTAATTTCTGCGATAGAGCTGGACCCCTCA
GTCTCTCAGGTATTGTACGAGGCGAAAGTGTGAAAGTTTTGTATGTGTTTTTATATGTACGACTGTATCGG
GAATTCCTTTAGCAAGAATCCTTTTAATACAGTATAATCTGTGCTACGGTACGTTACGTTCGCAGGGCACC
CGTTAATGTCTCATAGCCCAGACGATGGCGGATGGAAAGACATCATATTTTATTTTAATGCTGTCTTTATT
GCTGATTTATTTTGCTGTTTTTATTTGCTATTTTATATTTGCTAATTTTCATTATTGCGAAATATATTACA
TTGCTATTTTTATTATATACGCTAGATTCAATTTTATTTTTCCTATATTTTCAATTTAATTTTGATTTTGA
AGGTAAATATATATAATTAATTATTAAAAATGGCCTTCAGTTGTGGAACTCTCTCCTACTCTGCTGTCGCC
CAAGCTCCGTCCGTTGCCCATGCACCTCGTACATGGGAAGTTGATGAAGCCAGGCGGCGCCGAGTCATCAA
ACGTTTGGCGCTGGAGCAAGAACGTATTCGCAACGTTCTTGACGCCGGCGTCTATGACCAGGCGACATGGG
AACAGGAGGACGCGCGCGATAATGAGTTCCTAACGGAACAATTAAACAATTTATATACTATTTATTCGATC
GCCGAACGTTGCACGCGTCGACCTATTAAAGAGCACTCTCCTATATCAGTTTCGAATAGGTTTGCTCCATT
GGAATCCCTCAAAGTCGAGGTCGGTCAAGAAGCAGGCGAATGTATATTTAAGAAACCTAAATACACGCGCG
TTTGCAAGAAAGTGAAGCGTGTTGCAACCCGCTTCGTTCGTGAAAAAGTTGTTCGTCCTATGTGTTCTAGA
TCTCCTATGCTATTATTTAAGCTTAAGAAAATTATTTATGATTTGCACTTATATAGATTAAGAAAACAGAT
TAGGATTTTGAGACGTCAAAAACAGCGCGAGTATGAGTTAGAGTGTGTCACTAATCTGTTACAATTATCGA
ATCCGGTGCAGGCAAAACCAGAGATGGATAACCCTAATCCAGGAGGCGCGCCA***ATGGTGAGCAAGGGCGAG***
GAGCTGTTCACCGGGGTGGTGCCCATCCTGGTCGAGCTGGACGGCGACGTAAACGGCCACAAGTTCAGCGT
GTCCGGCGAGGGCGAGGGCGATGCCACCTACGGCAAGCTGACCCTGAAGTTCATCTGCACCACCGGCAAGC
TGCCCGTGCCCTGGCCCACCCTCGTGACCACCCTGACCTACGGCGTGCAGTGCTTCAGCCGCTACCCCGAC
CACATGAAGCAGCACGACTTCTTCAAGTCCGCCATGCCCGAAGGCTACGTCCAGGAGCGCACCATCTTCTT
CAAGGACGACGGCAACTACAAGACCCGCGCCGAGGTGAAGTTCGAGGGCGACACCCTGGTGAACCGCATCG
AGCTGAAGGGCATCGACTTCAAGGAGGACGGCAACATCCTGGGGCACAAGCTGGAGTACAACTACAACAGC
CACAACGTCTATATCATGGCCGACAAGCAGAAGAACGGCATCAAGGTGAACTTCAAGATCCGCCACAACAT
CGAGGACGGCAGCGTGCAGCTCGCCGACCACTACCAGCAGAACACCCCCATCGGCGACGGCCCCGTGCTGC
TGCCCGACAACCACTACCTGAGCACCCAGTCCGCCCTGAGCAAAGACCCCAACGAGAAGCGCGATCACATG
GTCCTGCTGGAGTTCGTGACCGCCGCCGGGATCACTCTCGGCATGGACGAGCTGTACAAGGGATCCGTGCA
GGCAAAACCAGAGATGGATAACCCTAATCCAGGACCTGATGGCGAGGGTGAAGTTGAATTAGAAAAGGATA
GTAATGTTGTTTTAACAACTCAGCGAGATCCTAGTACATCTATTCCAGCGCCGGTGAGCGTAAAATGGAGT
AGATGGACTAGTAATGATGTAGTAGATGATTACGCCACAATCACATCTCGATGGTATCAGATTGCTGAATT
TGTTTGGTCGAAGGATGATCCATTTGATAAGGAGTTAGCACGTTTAATTTTGCCTCGAGCTTTGTTATCTA
GTATAGAGGCTAATTCTGATGCTATATGTGATGTGCCTAATACTATTCCATTTAAGGTACACGCATATTGG
CGAGGCGATATGGAAGTTAGAGTTCAAATTAATTCAAATAAATTCCAAGTTGGTCAATTACAAGCTACTTG
GTATTATTCGGATCATGAGAATTTGAATATATCGTCTAAGAGAAGCGTTTATGGATTTTCACAAATGGATC
ATGCTTTGATTAGTGCGTCAGCAAGTAATGAAGCAAAATTAGTTATTCCATATAAGCATGTTTATCCATTT
TTACCAACAAGAATTGTGCCAGATTGGACTACTGGCATTTTAGATATGGGTGCTTTGAACATTCGTGTAAT
TGCTCCCTTACGGATGAGTGCTACTGGTCCAACTACCTGTAATGTCGTCGTGTTTATTAAATTAAATAACA
GCGAGTTTACAGGGACTTCCTCTGGTAAGTTTTATGCGAGCCAAATCAGGGCAAAACCTGAGATGGATCGT
ATATTAAATTTAGCAGAAGGATTGTTGAATAACACGATTGGTGGTAATAATATGGATAATCCTTCTTATCA
ACAATCTCCTCGTCATTTTGTCCCGACTGGTATGCACAGCTTAGCTTTAGGTACTAATTTAGTTGAACCAT
TACATGCATTACGTTTGGATGCAGCCGGTACGACACAACATCCTGTAGGTTGTGCTCCTGATGAAGATATG

ACTGTGTCTTCCATTGCATCTCGATATGGACTAATTAGACGGGTACAATGGAAGAAAGATCATGCTAAAGG
ATCGCTTTTGTTACAATTAGATGCTGATCCATTTGTGGAGCAAAGAATTGAAGGTACGAATCCAATATCTT
TGTATTGGTTCGCACCCGTGGGTGTAGTATCTAGTATGTTTATGCAATGGCGTGGTTCATTAGAATATAGG
TTTGATATTATAGCATCCCAATTTCATACTGGTAGGTTAATTGTAGGTTATGTGCCCGGTTTGACAGCATC
TTTGCAACTTCAAATGGACTATATGAAATTGAAGTCATCGAGTTATGTAGTATTTGATTTACAGGAAAGTA
ATAGCTTCACTTTTGAGGTGCCATATGTTTCATATAGACCATGGTGGGTGCGTAAATATGGTGGCAATTAT
TTGCCCTCGTCAACTGATGCTCCTAGTACATTATTCATGTATGTGCAGGTTCCGTTGATACCTATGGAAGC
TGTTTCAGATACTATTGATATCAATGTGTACGTACGGGGCGGTAGTTCATTTGAAGTTTGTGTTCCAGTCC
AACCTAGTTTAGGTTTGAATTGGAACACAGACTTTATTTTACGTAATGACGAAGAATACAGGGCTAAGACA
GGTTATGCACCATATTATGCTGGAGTGTGGCATAGCTTTAATAATAGTAATTCTCTTGTTTTTAGGTGGGG
ATCTGCTTCTGACCAAATTGCTCAGTGGCCGACAATTTCAGTACCAAGAGGTGAGTTAGCTTTCTTACGAA
TTAAGGATGGAAAGCAAGCTGCTGTAGGAACTCAACCTTGGCGTACGATGGTTGTTTGGCCTTCTGGTCAC
GGCTATAATATTGGTATACCCACGTATAATGCTGAACGAGCTCGCCAGCTTGCACAACACTTATATGGTGG
TGGATCATTAACTGATGAGAAGGCCAAACAATTATTTGTTCCTGCTAATCAACAAGGACCTGGTAAGGTAA
GTAATGGAAATCCGGTATGGGAAGTCATGCGTGCACCATTGTCAACACAGCGTGCGCATATGCAAGATTTT
GAATTTATTGAGGCTATTCCAGAAGGAGAGGAGTCTCGTAATACTACAGTCTTGGATACGACCACTACTTT
ACAGTCGAGTGGGTTTGGTCGCGCCTTCTTTGGAGAAGCTTTTAATGATCTTAAAACGTTAATGCGGCGAT
ATCAATTATATGGTCAATTATTATTGTCCGTTACTACGGATAAGGATATTGATCATTGTATGTTTACCTTC
CCTTGTTTACCACAAGGGTTAGCGTTAGACATTGGTTCTGCTGGCTCTCCACATGAAATCTTTAATAGATG
TCGTGATGGTATTATACCATTAATTGCATCTGGATATAGATTTTATAGAGGAGATTTGCGTTATAAGATTG
TTTTTCCAAGTAATGTTAATAGCAACATTTGGGTACAACATCGACCGGATCGTAGACTGGAAGGATGGTCC
GCGGCTAAGATTGTAAATTGTGATGCTGTGTCTACTGGTCAAGGAGTGTATAATCATGGTTATGCTAGTCA
CATTCAAATCACGCGTGTAAATAATGTTATAGAATTGGAAGTTCCATTTTATAATGCTACTTGTTATAATT
ATTTACAGGCGTTTAATGCGTCTAGCGCTGCATCTAGTTATGCAGTATCTTTAGGAGAAATATCGGTTGGT
TTTCAAGCCACAAGTGATGATATCGCATCTATCGTCAACAAACCTGTTACTATTTATTATAGTATTGGAGA
TGGTATGCAGTTTTCTCAGTGGGTTGGATATCAACCGATGATGATCCTAGATCAGCTTCCTGCACCAGTAG
TAAGGGCCGTGCCTGAGGGCCCTATTGCGAAGATTAAAAACTTCTTCCATCAAACAGCCGACGAAGTTAGA
GAAGCTCAGGCAGCAAAGATGCGTGAAGATATGGGTATGGTTGTTCAAGATGTTATTGGAGAACTTAGCCA
GGCCATACCGGATCTTCAACAACCGGAGGTTCAAGCAAATGTCTTCTCACTGGTGTCTCAGTTAGTGCATG
CTATTATAGGTACTAGTTTGAAGACAGTAGCTTGGGCGATTGTTTCGATTTTTGTGACTTTAGGATTAATT
GGACGCGAAATGATGCATTCAGTTATTACTGTAGTCAAGCGGTTACTTGAGAAATATCACTTGGCGACGCA
ACCCCAGGAATCCGCCAGTTCAAGTACTGTTATATCTGCCGTTCCAGAAGCTCCCAATGCTGAAGCGGAGG
AGGCAAGTGCTTGGGTATCCATTATTTATAATGGTGTGTGTAATATGCTTAATGTGGCTGCTCAAAAACCG
AAACAATTTAAAGATTGGGTAAAATTAGCTACTGTAGATTTTAGTAATAATTGTAGAGGTAGTAATCAGGT
ATTTGTGTTTTTCAAGAACACATTTGAAGTGTTGAAGAAAATGTGGGGTTATGTATTTTGTCAGAGTAATC
CTGCAGCGCGTTTGTTGAAAGCTGTGAATGACGAGCCTGAGATTTTGAAAGCATGGGTGAAGGAATGTCTG
TATTTGGATGATCCCAAATTTAGAATGCGTCGAGCGCATGATCAAGAGTATATCGAGCGAGTGTTTGCGGC
ACATTCATATGGACAAATTTTGCTACATGATTTAACTGCTGAAATGAACCAATCACGAAATTTGAGTGTGT
TTACACGTGTGTATGATCAAATTTCAAAATTGAAGACTGATCTTATGGAAATGGGATCGAATCCATATATA
AGGCGTGAATGTTTTACGATATGCATGTGTGGTGCATCTGGAATTGGAAAATCATATTTGACCGATTCTTT
ATGCAGCGAGCTCTTACGTGCGAGTCGTACTCCTGTGACAACAGGCATAAAATGTGTTGTTAATCCATTAT
CTGATTATTGGGATCAGTGTGATTTTCAGCCTGTTTTGTGCGTTGATGATATGTGGAGTGTTGAAACATCT
ACTACGCTCGATAAGCAGTTGAACATGCTTTTCCAGGTCCATTCCCCTATCGTGCTTTCTCCTCCTAAAGC
TGATTTAGAAGGTAAGAAAATGCGATATAACCCGGAAATATTTATATACAATACGAATAAGCCTTTCCCGA
GGTTTGATCGTATTGCTATGGAAGCTATTTATCGGCGTAGAAATGTTTTGATTGAATGTAAAGCGAGTGAA
GAGAAGAAGCGAGGATGCAAACATTGTGAGAACGATATTCCTATTGCTGAATGTAGTCCTAAGATGTTGAA
AGATTTCCATCACATCATATTTAGGTATGCACATGATGTATGTAATTCTGAGACCACATGGTCTGAATGGA
TGACGTATAATGAATTTCTTGAATGGATAACTCCTGTGTATATGGCTAACCGTCGTAAGGCGAATGAATCG
TTTAAGATGCGTGTAGATGAAATGCAAATGTTACGTATGGATGAGCCATTGGAAGGTGATAATATCCTCAA
TAAGTATGTTGAAGTTAATCAGCGCTTAGTGGAGGAAATGAAGGCATTTAAGGAGCGTACACTATGGTCAG
ATTTACATCGTTTAGGTGCGGAAGTTAGTGCGTCAGTTAAGAAAGCTTTACCAACTATTTCTATAACTGAA
AAACTACCACATTGGACTGTTCAATGTGGTATTGCTAAACCTGAGATGGATCATGCTTATGAGGTTATGAG
TTCGTATGCAGCTGGAATGAATGCAGAGATCGAAGCGCATGAACAAGTTCGGCGTTCATCAGTGGAATGTC
AATATGCAGAGCCTCAAGCTCCAAGAAATCCTGATGATGAAGGACCAACCATAGATGAAGAACTTATGGGC
GACACTGAATTCACATCACAGGCTCTAGAACGTCTTGTGGATGAAGGTTATATAACTGGAAAACAGAAGAA
GTATATAGCTACGTGGTGTAGTAAGCGTCGTGAACATACTGCTGACTTTGATCTTGTGTGGACTGATAATT
TGCGTGTGTTAAGCGCGTATGTGCATGAACGTTCATCTTCAACTCGGCTTTCTACGGATGACGTCAAGTTA

TATAAAACGATTAGCATGTTACATCAAAAGTACGATACCACAGAGTGTGCTAAATGTCAACATTGGTATGC
ACCGTTGACTGATATCTATGTTGATGACAAGAAATTGTTCTGGTGTCAGAAAGAGAAAAAGACACTTATTG
ATGTCCGAAAATTGTCGAAAGAAGATGTAACTGTTCAATCGAAATTGATTAATTTATCTGTTCCTTGTGGT
GAAGTATGTATGTTACATTCAAAATATTTTAATTATCTTTTCCATAAAGCATGGTTGTTCGAGAATCCAAC
TTGGCGCCTAATATATAATGGTACCAAGAAGGGTATGCCTGAGTACTTTATGAATTGTGTGGATGAAATAT
CGTTAGATTCCAAATTTGGTAAAGTGAAAGTATGGTTGCAAGCAATCATTGATAAGTATTTAACTCGTCCC
GTGAAAATGATTCGTGATTTTCTTTTCAAGTGGTGGCCGCAAGTTGCGTATGTGTTGAGCTTGCTAGGTAT
AATTGGTATAACTGCGTATGAAATGAGAAATCCGAAACCAACTTCTGAGGAATTAGCTGATCATTATGTGA
ATAGGCATTGTAGCTCTGATTTTTGGTCACCAGGACTGGCATCACCTCAAGGATTGAAATATAGTGAAGCA
GTAACAGTAAAGGCACCTAGAATCCATAGATTGCCAGTTACTACTAAGCCTCAGGGATCAACACAACAAGT
AGACGCCGCTGTGAATAAAATTCTACAGAACATGGTTTATATTGGTGTTGTTTTCCCAAAAGTGCCTGGTA
GTAAGTGGCGAGATATTAATTTTAGGTGTCTTATGCTTCATAATAGGCAATGTTTAATGTTGAGGCATTAT
ATTGAGTCAACTGCCGCCTTTCCTGAGGGAACCAAGTACTATTTTAAGTACATTCATAATCAAGAGACTAG
AATGTCTGGTGATATTTCTGGTATTGAAATTGATTTGTTGAATTTACCTAGATTGTATTATGGTGGTCTCG
CGGGAGAGGAGTCGTTTGATAGCAATATCGTGCTTGTGACTATGCCTAATCGTATTCCTGAGTGTAAGAGC
ATTATTAAATTTATAGCGTCACATAATGAGCATATACGTGCTCAGAATGATGGAGTGTTAGTAACTGGCGA
CCATACTCAGCTATTGGCTTTCGAGAATAATAATAAAACTCCAATAAGTATTAACGCTGATGGTTTGTATG
AGGTTATACTTCAAGGAGTATATACTTATCCATACCACGGCGATGGTGTTTGTGGTTCGATATTGTTATCT
CGGAATTTACAACGGCCAATTATAGGTATCCATGTTGCTGGTACTGAGGGATTGCATGGTTTTGGAGTCGC
TGAACCACTTGTACATGAAATGTTCACCGGTAAAGCGATCGAGAGTGAAAGAGAGCCGTATGATCGTGTGT
ATGAACTTCCGTTGCGTGAATTAGATGAATCTGATATTGGTTTAGATACCGATTTATATCCGATTGGTAGA
GTGGATGCAAAGCTAGCTCATGCTCAAAGCCCTTCTACTGGGATTAAAAAGACGCTTATTCATGGAACATT
TGATGTAAGGACTGAACCAAATCCGATGTCATCACGTGATCCAAGAATAGCGCCGCATGATCCTTTGAAGT
TAGGGTGTGAAAAGCATGGCATGCCTTGTTCACCGTTTAATAGGAAACATCTGGAATTAGCGACAAATCAT
TTGAAAGAAAAATTAGTTTCTGTAGTTAAACCAATAAATGGTTGCAAGATTAGAAGTTTGCAAGATGCTGT
ATGTGGTGTGCCTGGATTAGATGGGTTTGATTCGATATCGTGGAATACTAGTGCTGGTTTTCCTTTGTCTT
CATTAAAGCCACCTGGAACATCAGGTAAGCGATGGTTGTTTGATATTGAGCTACAAGACTCGGGATGTTAT
CTCCTGCGTGGAATGCGTCCCGAACTTGAGATTCAATTATCAACGACACAGTTAATGAGGAAAAAGGGAAT
AAAACCTCACACTATATTCACGGATTGTTTGAAAGATACTTGTTTGCCTGTTGAAAAATGTAGAATACCTG
GTAAGACTAGAATATTTAGTATAAGTCCGGTACAGTTTACCATACCGTTTCGACAGTATTACCTAGACTTT
ATGGCATCCTATCGAGCTGCACGACTTAATGCTGAGCATGGTATTGGTATTGATGTTAACAGCTTAGAGTG
GACAAATTTGGCAACAAGGTTGTCTAAGTATGGCACTCACATCGTGACAGGAGACTATAAGAATTTTGGTC
CTGGGTTAGATTCCGATGTTGCAGCTTCAGCGTTTGAAATTATTATCGACTGGGTATTACATTACACCGAA
GAAGATAATAAAGACGAAATGAAGCGAGTAATGTGGACCATGGCGCAAGAGATCTTAGCGCCTAGTCATCT
ATATCGCGATTTAGTGTACCGAGTACCTTGCGGAATTCCATCAGGTTCTCCAATAACGGACATATTGAATA
CAATTTCAAATTGTTTGTTAATTAGGTTAGCTTGGTTAGGTATTACTGATTTGCCTTTGTCCGAGTTCTCT
CAAAATGTTGTTCTTGTCTGTTATGGCGACGATCTTATCATGAATGTTAGCGATAACATGATTGATAAGTT
TAACGCCGTGACGATAGGAAAATTCTTTTCACAATATAAGATGGAATTTACGGATCAGGATAAATCAGGAA
ATACTGTAAAGTGGCGGACGTTACAGACTGCTACTTTCTTAAAACATGGGTTTTTAAAACATCCAACTAGA
CCTGTGTTTCTAGCTAACCTGGATAAGGTCTCGGTAGAAGGAACGACGAATTGGACTCATGCTCGAGGATT
GGGTCGTCGTACAGCAACCATAGAAAATGCTAAACAAGCGTTAGAGTTAGCATTTGGGTGGGGTCCTGAAT
ATTTTAATTACGTCAGAAATACCATTAAAATGGCTTTTGACAAGTTGGGTATTTATGAGGACCTTATCACA
TGGGATGAAATGGATGTTAGATGTTACGCTAGTGCGTAATATTTAATTTTAAATACTTATTAATTTTAATT
TTATTTTAGGTTATTGGAATTGAGGGAAGTACCACCCCCCAAGACCTTCGTTTTAAATCTACTAAGAGGAG
TAAACCTATATATAAGAGTCTAAAGACAGAGTGGATTAGACCATCATCTTTAGCTTATATATGGGGAAGGT
TGAGTTGCCTCTAAAGACTCAGCTCCGTAGTAGAGTAGTTTAATTACGATTAAAGTGGTACTCTAGGTTA
GGTGTTACTCGCGTATTATCAACTTGTGGTAATGCGTCCTAATTTTAGTATAGTTTTAACCATAATAGTAA
AAAAAAAAAAAAAAAAAAAAAAAAA

>DWV-S-DWV
TTTAAAATTCGCTATGGGAGGCGATTTATGCCTTCCATAGCGAATTACGGTGCAACTAACAATTTTAGATA
GTAGCCATGAACAAACATTATAGTGGCTCACTACGTATTGATCATTTTTATAATGACTTGCGTAGCATGAA
GCGCATGCTTGTAGTTGTAACTATGTTACTTTGCAAGTTGGAGTTTACTATTTTGGATTATGAATATGTGC
ACTTAGTGTCTGTATTTATAGTCGTTTGTGGTTCAAGGTTTTGTGTTAGTAGTACACTTATGTATGAATGT
ACCTTTAGTATGAATGTTATAGAATGACAATATCGAAAGAAAAATCTTTATAAAATACAAAAATATTGTTT
TTATTATTTCGATATGGTGTTTTATAGAGTAGATTGCCATGTGACCGCTCGTAGAAGTCCATTATGGTTTA

TCAATCGAAGTTGAATGTATTTATAAGAATATTATACTTAATTAATAATATTAGTAGTCCGTAACTATTAT
CATCCTTTTTCAGTTTGATGTGATAATAGACCACTGCAGTATCGAGTAGAGTTTCGAATGCGTAGTGCAAT
AGTACAATCACTGTCACCGACCATCTATTGTAATGATAGATTTGTCGGAAACCATTATTTATGAAGTGACT
AGCAATCATGGATTAAATTAGATGGTATTCTAGTTTAGAGGTGATTCGGCGCTGCGGTGCGACTGAAACTT
CTAAATTAGCATGTCAGATTGTATTATGAATGCGTTAGTAGTAATTTCTGCGATAGAGCTGGGACCCCTCA
GTCTCTCAGGTATTGTACGAGGCGAAAGTGTGAAAGTTTTGTATGTGTTTTTATATGTACGACTGTATCGG
GAATTCCTTTAGCAAGAATCCTTTTAATACAGTATAATCTGTGCTACGGTACGTTACGTTCGCAGGGCACC
CGTTAATGTCTCATAGCCCAGACGATGGCGGATGGAAAGACATCATATTTTATTTTAATGCTGTCTTTATT
GCTGATTTATTTTGCTGTTTTTATTTGCTATTTTATATTTGCTAATTTTCATTATTGCGAAATATATTACA
TTGCTATTTTTATTATATACGCTAGATTCAATTTTATTTTTCCTATATTTTCAATTTAATTTTGATTTTGA
AGGTAAATATATATAATTAATTATTAAAAATGGCCTTCAGTTGTGGAACTCTCTCCTACTCTGCTGTCGCC
CAAGCTCCGTCCGTTGCCCATGCACCTCGTACATGGGAAGTTGATGAAGCCAGGCGGCGCCGAGTCATCAA
ACGTTTGGCGCTGGAGCAAGAACGTATTCGCAACGTTCTTGACGCCGGCGTCTATGACCAGGCGACATGGG
AACAGGAGGACGCGCGCGATAATGAGTTCCTAACGGAACAATTAAACAATTTATATACTATTTATTCGATC
GCCGAACGTTGCACGCGTCGACCTATTAAAGAGCACTCTCCTATATCAGTTTCGAATAGGTTTGCTCCATT
GGAATCCCTCAAAGTCGAGGTCGGTCAAGAAGCAGGCGAATGTATATTTAAGAAACCTAAATACACGCGCG
TTTGCAAGAAAGTGAAGCGTGTTGCAACCCGCTTCGTTCGTGAAAAAGTTGTTCGTCCTATGTGTTCTAGA
TCTCCTATGCTATTATTTAAGCTTAAGAAAATTATTTATGATTTGCACTTATATAGATTAAGAAAACAGAT
TAGGATTTTGAGACGTCAAAAACAGCGCGAGTATGAGTTAGAGTGTGTCACTAATCTGTTACAATTATCGA
ATCCGGTGCAGGCAAAACCAGAG**GGCGCGCCA*****ATGGTGAGCAAGGGCGAGGAGCTGTTCACCGGGGTGGTG***
CCCATCCTGGTCGAGCTGGACGGCGACGTAAACGGCCACAAGTTCAGCGTGTCCGGCGAGGGCGAGGGCGA
TGCCACCTACGGCAAGCTGACCCTGAAGTTCATCTGCACCACCGGCAAGCTGCCCGTGCCCTGGCCCACCC
TCGTGACCACCCTGACCTACGGCGTGCAGTGCTTCAGCCGCTACCCCGACCACATGAAGCAGCACGACTTC
TTCAAGTCCGCCATGCCCGAAGGCTACGTCCAGGAGCGCACCATCTTCTTCAAGGACGACGGCAACTACAA
GACCCGCGCCGAGGTGAAGTTCGAGGGCGACACCCTGGTGAACCGCATCGAGCTGAAGGGCATCGACTTCA
AGGAGGACGGCAACATCCTGGGGCACAAGCTGGAGTACAACTACAACAGCCACAACGTCTATATCATGGCC
GACAAGCAGAAGAACGGCATCAAGGTGAACTTCAAGATCCGCCACAACATCGAGGACGGCAGCGTGCAGCT
CGCCGACCACTACCAGCAGAACACCCCCATCGGCGACGGCCCCGTGCTGCTGCCCGACAACCACTACCTGA
GCACCCAGTCCGCCCTGAGCAAAGACCCCAACGAGAAGCGCGATCACATGGTCCTGCTGGAGTTCGTGACC
GCCGCCGGGATCACTCTCGGCATGGACGAGCTGTACAAGGGATCCGTGCAGGCAAAACCAGAGATGGATAA
CCCTAATCCAGGACCTGATGGCGAGGGTGAAGTTGAATTAGAAAAGGATAGTAATGTTGTTTTAACAACTC
AGCGAGATCCTAGTACATCTATTCCAGCGCCGGTGAGCGTAAAATGGAGTAGATGGACTAGTAATGATGTA
GTAGATGATTACGCCACAATCACATCTCGATGGTATCAGATTGCTGAATTTGTTTGGTCGAAGGATGATCC
ATTTGATAAGGAGTTAGCACGTTTAATTTTGCCTCGAGCTTTGTTATCTAGTATAGAGGCTAATTCTGATG
CTATATGTGATGTGCCTAATACTATTCCATTTAAGGTACACGCATATTGGCGAGGCGATATGGAAGTTAGA
GTTCAAATTAATTCAAATAAATTCCAAGTTGGTCAATTACAAGCTACTTGGTATTATTCGGATCATGAGAA
TTTGAATATATCGTCTAAGAGAAGCGTTTATGGATTTTCACAAATGGATCATGCTTTGATTAGTGCGTCAG
CAAGTAATGAAGCAAAATTAGTTATTCCATATAAGCATGTTTATCCATTTTTACCAACAAGAATTGTGCCA
GATTGGACTACTGGCATTTTAGATATGGGTGCTTTGAACATTCGTGTAATTGCTCCCTTACGGATGAGTGC
TACTGGTCCAACTACCTGTAATGTCGTCGTGTTTATTAAATTAAATAACAGCGAGTTTACAGGGACTTCCT
CTGGTAAGTTTTATGCGAGCCAAATCAGGGCAAAACCTGAGATGGATCGTATATTAAATTTAGCAGAAGGA
TTGTTGAATAACACGATTGGTGGTAATAATATGGATAATCCTTCTTATCAACAATCTCCTCGTCATTTTGT
CCCGACTGGTATGCACAGCTTAGCTTTAGGTACTAATTTAGTTGAACCATTACATGCATTACGTTTGGATG
CAGCCGGTACGACACAACATCCTGTAGGTTGTGCTCCTGATGAAGATATGACTGTGTCTTCCATTGCATCT
CGATATGGACTAATTAGACGGGTACAATGGAAGAAAGATCATGCTAAAGGATCGCTTTTGTTACAATTAGA
TGCTGATCCATTTGTGGAGCAAAGAATTGAAGGTACGAATCCAATATCTTTGTATTGGTTCGCACCCGTGG
GTGTAGTATCTAGTATGTTTATGCAATGGCGTGGTTCATTAGAATATAGGTTTGATATTATAGCATCCCAA
TTTCATACTGGTAGGTTAATTGTAGGTTATGTGCCCGGTTTGACAGCATCTTTGCAACTTCAAATGGACTA
TATGAAATTGAAGTCATCGAGTTATGTAGTATTTGATTTACAGGAAAGTAATAGCTTCACTTTTGAGGTGC
CATATGTTTCATATAGACCATGGTGGGTGCGTAAATATGGTGGCAATTATTTGCCCTCGTCAACTGATGCT
CCTAGTACATTATTCATGTATGTGCAGGTTCCGTTGATACCTATGGAAGCTGTTTCAGATACTATTGATAT
CAATGTGTACGTACGGGGCGGTAGTTCATTTGAAGTTTGTGTTCCAGTCCAACCTAGTTTAGGTTTGAATT
GGAACACAGACTTTATTTTACGTAATGACGAAGAATACAGGGCTAAGACAGGTTATGCACCATATTATGCT
GGAGTGTGGCATAGCTTTAATAATAGTAATTCTCTTGTTTTTAGGTGGGGATCTGCTTCTGACCAAATTGC
TCAGTGGCCGACAATTTCAGTACCAAGAGGTGAGTTAGCTTTCTTACGAATTAAGGATGGAAAGCAAGCTG
CTGTAGGAACTCAACCTTGGCGTACGATGGTTGTTTGGCCTTCTGGTCACGGCTATAATATTGGTATACCC
ACGTATAATGCTGAACGAGCTCGCCAGCTTGCACAACACTTATATGGTGGTGGATCATTAACTGATGAGAA

GGCCAAACAATTATTTGTTCCTGCTAATCAACAAGGACCTGGTAAGGTAAGTAATGGAAATCCGGTATGGG
AAGTCATGCGTGCACCATTGTCAACACAGCGTGCGCATATGCAAGATTTTGAATTTATTGAGGCTATTCCA
GAAGGAGAGGAGTCTCGTAATACTACAGTCTTGGATACGACCACTACTTTACAGTCGAGTGGGTTTGGTCG
CGCCTTCTTTGGAGAAGCTTTTAATGATCTTAAAACGTTAATGCGGCGATATCAATTATATGGTCAATTAT
TATTGTCCGTTACTACGGATAAGGATATTGATCATTGTATGTTTACCTTCCCTTGTTTACCACAAGGGTTA
GCGTTAGACATTGGTTCTGCTGGCTCTCCACATGAAATCTTTAATAGATGTCGTGATGGTATTATACCATT
AATTGCATCTGGATATAGATTTTATAGAGGAGATTTGCGTTATAAGATTGTTTTTCCAAGTAATGTTAATA
GCAACATTTGGGTACAACATCGACCGGATCGTAGACTGGAAGGATGGTCCGCGGCTAAGATTGTAAATTGT
GATGCTGTGTCTACTGGTCAAGGAGTGTATAATCATGGTTATGCTAGTCACATTCAAATCACGCGTGTAAA
TAATGTTATAGAATTGGAAGTTCCATTTTATAATGCTACTTGTTATAATTATTTACAGGCGTTTAATGCGT
CTAGCGCTGCATCTAGTTATGCAGTATCTTTAGGAGAAATATCGGTTGGTTTTCAAGCCACAAGTGATGAT
ATCGCATCTATCGTCAACAAACCTGTTACTATTTATTATAGTATTGGAGATGGTATGCAGTTTTCTCAGTG
GGTTGGATATCAACCGATGATGATCCTAGATCAGCTTCCTGCACCAGTAGTAAGGGCCGTGCCTGAGGGCC
CTATTGCGAAGATTAAAAACTTCTTCCATCAAACAGCCGACGAAGTTAGAGAAGCTCAGGCAGCAAAGATG
CGTGAAGATATGGGTATGGTTGTTCAAGATGTTATTGGAGAACTTAGCCAGGCCATACCGGATCTTCAACA
ACCGGAGGTTCAAGCAAATGTCTTCTCACTGGTGTCTCAGTTAGTGCATGCTATTATAGGTACTAGTTTGA
AGACAGTAGCTTGGGCGATTGTTTCGATTTTTGTGACTTTAGGATTAATTGGACGCGAAATGATGCATTCA
GTTATTACTGTAGTCAAGCGGTTACTTGAGAAATATCACTTGGCGACGCAACCCCAGGAATCCGCCAGTTC
AAGTACTGTTATATCTGCCGTTCCAGAAGCTCCCAATGCTGAAGCGGAGGAGGCAAGTGCTTGGGTATCCA
TTATTTATAATGGTGTGTGTAATATGCTTAATGTGGCTGCTCAAAAACCGAAACAATTTAAAGATTGGGTA
AAATTAGCTACTGTAGATTTTAGTAATAATTGTAGAGGTAGTAATCAGGTATTTGTGTTTTTCAAGAACAC
ATTTGAAGTGTTGAAGAAAATGTGGGGTTATGTATTTTGTCAGAGTAATCCTGCAGCGCGTTTGTTGAAAG
CTGTGAATGACGAGCCTGAGATTTTGAAAGCATGGGTGAAGGAATGTCTGTATTTGGATGATCCCAAATTT
AGAATGCGTCGAGCGCATGATCAAGAGTATATCGAGCGAGTGTTTGCGGCACATTCATATGGACAAATTTT
GCTACATGATTTAACTGCTGAAATGAACCAATCACGAAATTTGAGTGTGTTTACACGTGTGTATGATCAAA
TTTCAAAATTGAAGACTGATCTTATGGAAATGGGATCGAATCCATATATAAGGCGTGAATGTTTTACGATA
TGCATGTGTGGTGCATCTGGAATTGGAAAATCATATTTGACCGATTCTTTATGCAGCGAGCTCTTACGTGC
GAGTCGTACTCCTGTGACAACAGGCATAAAATGTGTTGTTAATCCATTATCTGATTATTGGGATCAGTGTG
ATTTTCAGCCTGTTTTGTGCGTTGATGATATGTGGAGTGTTGAAACATCTACTACGCTCGATAAGCAGTTG
AACATGCTTTTCCAGGTCCATTCCCCTATCGTGCTTTCTCCTCCTAAAGCTGATTTAGAAGGTAAGAAAAT
GCGATATAACCCGGAAATATTTATATACAATACGAATAAGCCTTTCCCGAGGTTTGATCGTATTGCTATGG
AAGCTATTTATCGGCGTAGAAATGTTTTGATTGAATGTAAAGCGAGTGAAGAGAAGAAGCGAGGATGCAAA
CATTGTGAGAACGATATTCCTATTGCTGAATGTAGTCCTAAGATGTTGAAAGATTTCCATCACATCATATT
TAGGTATGCACATGATGTATGTAATTCTGAGACCACATGGTCTGAATGGATGACGTATAATGAATTTCTTG
AATGGATAACTCCTGTGTATATGGCTAACCGTCGTAAGGCGAATGAATCGTTTAAGATGCGTGTAGATGAA
ATGCAAATGTTACGTATGGATGAGCCATTGGAAGGTGATAATATCCTCAATAAGTATGTTGAAGTTAATCA
GCGCTTAGTGGAGGAAATGAAGGCATTTAAGGAGCGTACACTATGGTCAGATTTACATCGTTTAGGTGCGG
AAGTTAGTGCGTCAGTTAAGAAAGCTTTACCAACTATTTCTATAACTGAAAAACTACCACATTGGACTGTT
CAATGTGGTATTGCTAAACCTGAGATGGATCATGCTTATGAGGTTATGAGTTCGTATGCAGCTGGAATGAA
TGCAGAGATCGAAGCGCATGAACAAGTTCGGCGTTCATCAGTGGAATGTCAATATGCAGAGCCTCAAGCTC
CAAGAAATCCTGATGATGAAGGACCAACCATAGATGAAGAACTTATGGGCGACACTGAATTCACATCACAG
GCTCTAGAACGTCTTGTGGATGAAGGTTATATAACTGGAAAACAGAAGAAGTATATAGCTACGTGGTGTAG
TAAGCGTCGTGAACATACTGCTGACTTTGATCTTGTGTGGACTGATAATTTGCGTGTGTTAAGCGCGTATG
TGCATGAACGTTCATCTTCAACTCGGCTTTCTACGGATGACGTCAAGTTATATAAAACGATTAGCATGTTA
CATCAAAAGTACGATACCACAGAGTGTGCTAAATGTCAACATTGGTATGCACCGTTGACTGATATCTATGT
TGATGACAAGAAATTGTTCTGGTGTCAGAAAGAGAAAAAGACACTTATTGATGTCCGAAAATTGTCGAAAG
AAGATGTAACTGTTCAATCGAAATTGATTAATTTATCTGTTCCTTGTGGTGAAGTATGTATGTTACATTCA
AAATATTTTAATTATCTTTTCCATAAAGCATGGTTGTTCGAGAATCCAACTTGGCGCCTAATATATAATGG
TACCAAGAAGGGTATGCCTGAGTACTTTATGAATTGTGTGGATGAAATATCGTTAGATTCCAAATTTGGTA
AAGTGAAAGTATGGTTGCAAGCAATCATTGATAAGTATTTAACTCGTCCCGTGAAAATGATTCGTGATTTT
CTTTTCAAGTGGTGGCCGCAAGTTGCGTATGTGTTGAGCTTGCTAGGTATAATTGGTATAACTGCGTATGA
AATGAGAAATCCGAAACCAACTTCTGAGGAATTAGCTGATCATTATGTGAATAGGCATTGTAGCTCTGATT
TTTGGTCACCAGGACTGGCATCACCTCAAGGATTGAAATATAGTGAAGCAGTAACAGTAAAGGCACCTAGA
ATCCATAGATTGCCAGTTACTACTAAGCCTCAGGGATCAACACAACAAGTAGACGCCGCTGTGAATAAAAT
TCTACAGAACATGGTTTATATTGGTGTTGTTTTCCCAAAAGTGCCTGGTAGTAAGTGGCGAGATATTAATT
TTAGGTGTCTTATGCTTCATAATAGGCAATGTTTAATGTTGAGGCATTATATTGAGTCAACTGCCGCCTTT
CCTGAGGGAACCAAGTACTATTTTAAGTACATTCATAATCAAGAGACTAGAATGTCTGGTGATATTTCTGG

TATTGAAATTGATTTGTTGAATTTACCTAGATTGTATTATGGTGGTCTCGCGGGAGAGGAGTCGTTTGATA
GCAATATCGTGCTTGTGACTATGCCTAATCGTATTCCTGAGTGTAAGAGCATTATTAAATTTATAGCGTCA
CATAATGAGCATATACGTGCTCAGAATGATGGAGTGTTAGTAACTGGCGACCATACTCAGCTATTGGCTTT
CGAGAATAATAATAAAACTCCAATAAGTATTAACGCTGATGGTTTGTATGAGGTTATACTTCAAGGAGTAT
ATACTTATCCATACCACGGCGATGGTGTTTGTGGTTCGATATTGTTATCTCGGAATTTACAACGGCCAATT
ATAGGTATCCATGTTGCTGGTACTGAGGGATTGCATGGTTTTGGAGTCGCTGAACCACTTGTACATGAAAT
GTTCACCGGTAAAGCGATCGAGAGTGAAAGAGAGCCGTATGATCGTGTGTATGAACTTCCGTTGCGTGAAT
TAGATGAATCTGATATTGGTTTAGATACCGATTTATATCCGATTGGTAGAGTGGATGCAAAGCTAGCTCAT
GCTCAAAGCCCTTCTACTGGGATTAAAAAGACGCTTATTCATGGAACATTTGATGTAAGGACTGAACCAAA
TCCGATGTCATCACGTGATCCAAGAATAGCGCCGCATGATCCTTTGAAGTTAGGGTGTGAAAAGCATGGCA
TGCCTTGTTCACCGTTTAATAGGAAACATCTGGAATTAGCGACAAATCATTTGAAAGAAAAATTAGTTTCT
GTAGTTAAACCAATAAATGGTTGCAAGATTAGAAGTTTGCAAGATGCTGTATGTGGTGTGCCTGGATTAGA
TGGGTTTGATTCGATATCGTGGAATACTAGTGCTGGTTTTCCTTTGTCTTCATTAAAGCCACCTGGAACAT
CAGGTAAGCGATGGTTGTTTGATATTGAGCTACAAGACTCGGGATGTTATCTCCTGCGTGGAATGCGTCCC
GAACTTGAGATTCAATTATCAACGACACAGTTAATGAGGAAAAAGGGAATAAAACCTCACACTATATTCAC
GGATTGTTTGAAAGATACTTGTTTGCCTGTTGAAAAATGTAGAATACCTGGTAAGACTAGAATATTTAGTA
TAAGTCCGGTACAGTTTACCATACCGTTTCGACAGTATTACCTAGACTTTATGGCATCCTATCGAGCTGCA
CGACTTAATGCTGAGCATGGTATTGGTATTGATGTTAACAGCTTAGAGTGGACAAATTTGGCAACAAGGTT
GTCTAAGTATGGCACTCACATCGTGACAGGAGACTATAAGAATTTTGGTCCTGGGTTAGATTCCGATGTTG
CAGCTTCAGCGTTTGAAATTATTATCGACTGGGTATTACATTACACCGAAGAAGATAATAAAGACGAAATG
AAGCGAGTAATGTGGACCATGGCGCAAGAGATCTTAGCGCCTAGTCATCTATATCGCGATTTAGTGTACCG
AGTACCTTGCGGAATTCCATCAGGTTCTCCAATAACGGACATATTGAATACAATTTCAAATTGTTTGTTAA
TTAGGTTAGCTTGGTTAGGTATTACTGATTTGCCTTTGTCCGAGTTCTCTCAAAATGTTGTTCTTGTCTGT
TATGGCGACGATCTTATCATGAATGTTAGCGATAACATGATTGATAAGTTTAACGCCGTGACGATAGGAAA
ATTCTTTTCACAATATAAGATGGAATTTACGGATCAGGATAAATCAGGAAATACTGTAAAGTGGCGGACGT
TACAGACTGCTACTTTCTTAAAACATGGGTTTTTAAAACATCCAACTAGACCTGTGTTTCTAGCTAACCTG
GATAAGGTCTCGGTAGAAGGAACGACGAATTGGACTCATGCTCGAGGATTGGGTCGTCGTACAGCAACCAT
AGAAAATGCTAAACAAGCGTTAGAGTTAGCATTTGGGTGGGGTCCTGAATATTTTAATTACGTCAGAAATA
CCATTAAAATGGCTTTTGACAAGTTGGGTATTTATGAGGACCTTATCACATGGGATGAAATGGATGTTAGA
TGTTACGCTAGTGCGTAATATTTAATTTTAAATACTTATTAATTTTAATTTTATTTTAGGTTATTGGAATT
GAGGGAAGTACCACCCCCCAAGACCTTCGTTTTAAATCTACTAAGAGGAGTAAACCTATATATAAGAGTCT
AAAGACAGAGTGGATTAGACCATCATCTTTAGCTTATATATGGGGAAGGTTGAGTTGCCTCTAAAGACTCA
GCTCCGTAGTAGAGTAGTTTTAATTACGATTAAAGTGGTACTCTAGGTTAGGTGTTACTCGCGTATTATCA
ACTTGTGGTAATGCGTCCTAATTTTAGTATAGTTTTAACCATAATAGTAAAAAAAAAAAAAAAAAAAAAAA
AAAA
