# Supplementary material for: Development of a Honey Bee RNA Virus Vector Based on the Genome of a Deformed Wing Virus
Source: Viruses. 2020 Mar 28;12(4):374. doi: 10.3390/v12040374 (PMC7232236; doi:10.3390/v12040374)
Supplement: Supplementary file 1 [file viruses-12-00374-s001.zip › Figure_S3.pdf]

A

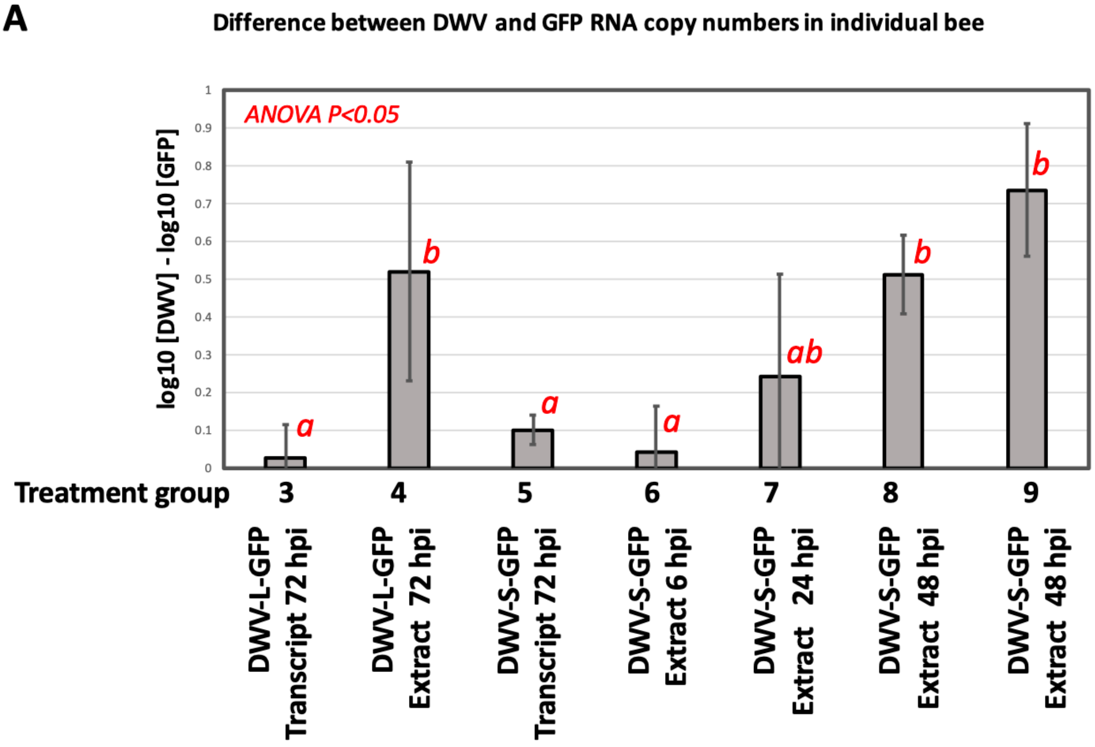

B

	**Ttreatment group***	**4**	**5**	**6**	**7**	**8**	**9**
**Ttreatment group ***		**DWV-L-GFP - Extract 72 hpi**	**DWV-S-GFP - Transcript 72 hpi**	**DWV-S-GFP - Extract 6 hpi**	**DWV-S-GFP - Extract 24 hpi**	**DWV-S-GFP - Extract 48 hpi**	**DWV-S-GFP - Extract 72 hpi**
**3**	**DWV-L-GFP -Transcript 72 hpi**	0.03824	**0.282417**	**0.868186**	**0.267553**	0.000228	0.000342
**4**	**DWV-L-GFP -Extract 72 hpi**		0.033378	0.012333	**0.127334**	**1.0000**	**0.157573**
**5**	**DWV-S-GFP -Transcript 72 hpi**			**0.446633**	**0.383298**	<.0001	0.000125
**6**	**DWV-S-GFP -Extract 6 hpi**				**0.194915**	<.0001	<.0001
**7**	**DWV-S-GFP -Extract 24 hpi**					0.041487	0.0028
**8**	**DWV-S-GFP -Extract 48 hpi**						0.019779

* Treatment group number as in Fig. 2

Figure S3. Accumulation of the *egfp* deletant genomes in individual honey bee pupae. (A) Average copy numbers for the differences between DWV and GFP loads (Log_{10}(DWV) - Log_{10}(GFP) calculated for individual pupa in the treatment groups are shown as grey graphs, the error bars show ± standard deviation (SD). Treatments are shown below the graphs. Inocula: PBS - phosphate buffered saline; “Transcript”, *in vitro* RNA transcript; Extract, filtered extract from the pupae infected with the corresponding RNA transcript. Red letters above the bars indicate significantly groups (ANOVA, $P < 0.05$). **(B)** Statistical significance of the Log_{10}(DWV) - Log_{10}(GFP) differences between the treatment groups. P-values, ANOVA analysis. Non-significant ($P>0.05\%$, ANOVA) are highlighted.
