# Supplementary material for: Development of a Honey Bee RNA Virus Vector Based on the Genome of a Deformed Wing Virus
Source: Viruses. 2020 Mar 28;12(4):374. doi: 10.3390/v12040374 (PMC7232236; doi:10.3390/v12040374)
Supplement: Supplementary file 1 [file viruses-12-00374-s001.zip › Figure_S2.pdf]

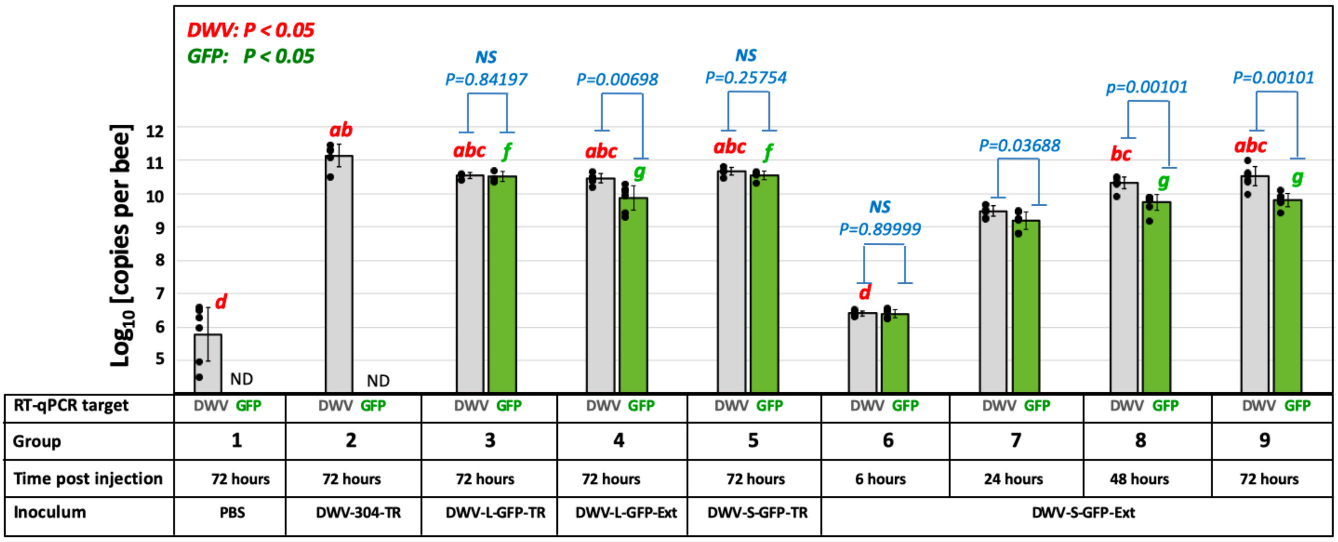

RT-qPCR target	DWV GFP	DWV GFP	DWV GFP	DWV GFP	DWV GFP	DWV GFP	DWV GFP	DWV GFP	DWV GFP
Group	1	2	3	4	5	6	7	8	9
Time post injection	72 hours	72 hours	72 hours	72 hours	72 hours	6 hours	24 hours	48 hours	72 hours
Inoculum	PBS	DWV-304-TR	DWV-L-GFP-TR	DWV-L-GFP-Ext	DWV-S-GFP-TR	DWV-S-GFP-Ext			

Figure S2. Replication of the GFP-tagged DWV in honey bee pupae analyzed using one-way ANOVA followed by Tukey post-hoc test. Average copy numbers of DWV and GFP RNA per pupa revealed by RT-qPCR are shown as the light grey and green graphs, respectively, the error bars show ± standard deviation (SD), black dots show DWV RNA or GFP RNA copy numbers in individual pupae. Treatments are shown below the graphs; Inocula: PBS - phosphate buffered saline; suffix -TR, in vitro RNA transcript; suffix -Ext, filtered extract from the pupae infected with the corresponding *in vitro* RNA transcript. Red letters above the bars indicate significantly and non-significantly different groups ($P < 0.01$). Blue bars – statistical significance of the DWV and GFP copy numbers within the same group NS - non significant, $P > 0.05$). Statistical analyses of viral data, log-transformed copy numbers of DWV and GFP RNA per pupa, were conducted by one-way ANOVA followed by Tukey post-hoc test to identify differences between the groups ($p < 0.05$).
