# Supplementary material for: Development of a Honey Bee RNA Virus Vector Based on the Genome of a Deformed Wing Virus
Source: Viruses. 2020 Mar 28;12(4):374. doi: 10.3390/v12040374 (PMC7232236; doi:10.3390/v12040374)
Supplement: Supplementary file 1 [file viruses-12-00374-s001.zip › Figure_S1.pdf]

A.

```
DWV-L-GFP          MAFSCGTLSYSAVAQAPSVAHAPRTWEVDEARRRRVIKRLALEQERIRNVLDAGVYDQAT 60
DWV-S-GFP          MAFSCGTLSYSAVAQAPSVAHAPRTWEVDEARRRRVIKRLALEQERIRNVLDAGVYDQAT 60
DWV-304-MG831200   MAFSCGTLSYSAVAQAPSVAHAPRTWEVDEARRRRVIKRLALEQERIRNVLDAGVYDQAT 60
                   ************************************************************

DWV-L-GFP          WEQEDARDNEFLTEQLNNLYTIYSIAERCTRRPIKEHSPISVSNRFAPLESLKVEVGQEA 120
DWV-S-GFP          WEQEDARDNEFLTEQLNNLYTIYSIAERCTRRPIKEHSPISVSNRFAPLESLKVEVGQEA 120
DWV-304-MG831200   WEQEDARDNEFLTEQLNNLYTIYSIAERCTRRPIKEHSPISVSNRFAPLESLKVEVGQEA 120
                   ************************************************************

DWV-L-GFP          GECIFKKPKYTRVCKKVKRVATRFVREKVVRPMCSRSPMLLFKLKKIIYDLHLYRLRKQI 180
DWV-S-GFP          GECIFKKPKYTRVCKKVKRVATRFVREKVVRPMCSRSPMLLFKLKKIIYDLHLYRLRKQI 180
DWV-304-MG831200   GECIFKKPKYTRVCKKVKRVATRFVREKVVRPMCSRSPMLLFKLKKIIYDLHLYRLRKQI 180
                   ************************************************************

DWV-L-GFP          RILRRQKQREYELECVTNLLQLSNPVQAKPEMDNPNPGGAPMVSKGEELFTGVVPILVEL 240
DWV-S-GFP          RILRRQKQREYELECVTNLLQLSNPVQAKPE-------GAPMVSKGEELFTGVVPILVEL 233
DWV-304-MG831200   RILRRQKQREYELECVTNLLQLSNPVQAKPE----------------------------- 209
                   *******************************

DWV-L-GFP          DGDVNGHKFSVSGEGEGDATYGKLTLKFICTTGKLPVPWPTLVTTLTYGVQCFSRYPDHM 300
DWV-S-GFP          DGDVNGHKFSVSGEGEGDATYGKLTLKFICTTGKLPVPWPTLVTTLTYGVQCFSRYPDHM 293
DWV-304-MG831200   ------------------------------------------------------------ 209

DWV-L-GFP          KQHDFFKSAMPEGYVQERTIFFKDDGNYKTRAEVKFEGDTLVNRIELKGIDFKEDGNILG 360
DWV-S-GFP          KQHDFFKSAMPEGYVQERTIFFKDDGNYKTRAEVKFEGDTLVNRIELKGIDFKEDGNILG 353
DWV-304-MG831200   ------------------------------------------------------------ 209

DWV-L-GFP          HKLEYNYNSHNVYIMADKQKNGIKVNFKIRHNIEDGSVQLADHYQQNTPIGDGPVLLPDN 420
DWV-S-GFP          HKLEYNYNSHNVYIMADKQKNGIKVNFKIRHNIEDGSVQLADHYQQNTPIGDGPVLLPDN 413
DWV-304-MG831200   ------------------------------------------------------------ 209

DWV-L-GFP          HYLSTQSALSKDPNEKRDHMVLLEFVTAAGITLGMDELYKGSVQAKPEMDNPNPGPDGEG 480
DWV-S-GFP          HYLSTQSALSKDPNEKRDHMVLLEFVTAAGITLGMDELYKGSVQAKPEMDNPNPGPDGEG 473
DWV-304-MG831200   ------------------------------------------------MDNPNPGPDGEG 223
                                                                   ************

DWV-L-GFP          EVELEKDSNVVLTTQRDPSTSIPAPVSVKWSRWTSNDVVDDYATITSRWYQIAEFVWSKD 540
DWV-S-GFP          EVELEKDSNVVLTTQRDPSTSIPAPVSVKWSRWTSNDVVDDYATITSRWYQIAEFVWSKD 533
DWV-304-MG831200   EVELEKDSNVVLTTQRDPSTSIPAPVSVKWSRWTSNDVVDDYATITSRWYQIAEFVWSKD 283
                   ************************************************************
```

B.

```
DWV-S-GFP          GAPMVSKGEELFTGVVPILVELDGDVNGHKFSVSGEGEGDATYGKLTLKFICT     53
DWV-L-GFP   MDNPNPGGAPMVSKGEELFTGVVPILVELDGDVNGHKFSVSGEGEGDATYGKLTLKFICT     60
Plant-GFP             MASKGEELFTGVVPILVELDGDVNGHKFSVSGEGEGDATYGKLTLKFICT     50
                      *.************************************************

DWV-S-GFP   TGKLPVPWPTLVTTLTYGVQCFSRYPDHMKQHDFFKSAMPEGYVQERTIFFKDDGNYKTR    113
DWV-L-GFP   TGKLPVPWPTLVTTLTYGVQCFSRYPDHMKQHDFFKSAMPEGYVQERTIFFKDDGNYKTR    120
Plant-GFP   TGKLPVPWPTLVTTFSYGVQCFSRYPDHMKRHDFFKSAMPEGYVQERTIFFKDDGNYKTR    110
            **************::**************:*****************************

DWV-S-GFP   AEVKFEGDTLVNRIELKGIDFKEDGNILGHKLEYNYNSHNVYIMADKQKNGIKVNFKIRH    173
DWV-L-GFP   AEVKFEGDTLVNRIELKGIDFKEDGNILGHKLEYNYNSHNVYIMADKQKNGIKVNFKIRH    180
Plant-GFP   AEVKFEGDTLVNRIELKGIDFKEDGNILGHKLEYNYNSHNVYITADKQKNGIKANFKIRH    170
            ******************************************* *********.******

DWV-S-GFP   NIEDGSVQLADHYQQNTPIGDGPVLLPDNHYLSTQSALSKDPNEKRDHMVLLEFVTAAGI    233
DWV-L-GFP   NIEDGSVQLADHYQQNTPIGDGPVLLPDNHYLSTQSALSKDPNEKRDHMVLLEFVTAAGI    240
Plant-GFP   NIEDGSVQLADHYQQNTPIGDGPVLLPDNHYLSTQSALSKDPNEKRDHMVLLEFVTAAGI    230
            ************************************************************

DWV-S-GFP   TLGMDELYKGSVQAKPE  250
DWV-L-GFP   TLGMDELYKGSVQAKPE  257
Plant-GFP   THGMDELYK          239
            * *******
```

Figure S1. The eGFP-coding sequences inserts in DWV vector. (A) Alignment of the N-terminal sections of the polyproteins encoded by infectious cDNA constructs DWV-L-GFP, DWV-S-GFP, and DWV-304 (GenBank accession number MG831200). **(B)** Alignment of the eGFP peptides generated in the honey bee pupae by the DWV 3C proteolytic cleavage of the polyproteins of DWV-S-GFP (27.97 kDa) and DWV-L-GFP (28.69 kDa), and free GFP control expressed in plants (26.93 kDa). Peptide recognized by polyclonal anti-GFP antibodies is underscored. Yellow highlight – parts the DWV LP-VP1 proteolytic cleavage peptide. Blue highlight – amino acid residues introduced by insertion of *Asc*I and *Bam*HI restriction sites.
